# Biosafety of Mesoporous Silica Nanoparticles

**DOI:** 10.3390/biomimetics3030022

**Published:** 2018-08-15

**Authors:** Estelle Rascol, Cédric Pisani, Christophe Dorandeu, Jeff L. Nyalosaso, Clarence Charnay, Morgane Daurat, Afitz Da Silva, Jean-Marie Devoisselle, Jean-Charles Gaillard, Jean Armengaud, Odette Prat, Marie Maynadier, Magali Gary-Bobo, Marcel Garcia, Joël Chopineau, Yannick Guari

**Affiliations:** 1 Institute of Chemistry and Biology of Membranes and Nano-objects (CBMN) UMR-5248, CNRS, University of Bordeaux, INP, Allée Geoffroy St Hilaire, 33600 Pessac, France; 2 Institute Charles Gerhardt of Montpellier (ICGM), Place E. Bataillon, 34095 Montpellier, France; christophe.dorandeu@umontpellier.fr (C.D.); jeff.nyalosaso@gmail.com (J.L.N.); clarence.charnay@univ-montp2.fr (C.C.); jm.devoisselle@univ-montp1.fr (J.-M.D.); joel.chopineau@enscm.fr (J.C.); yannick.guari@univ-montp2.fr (Y.G.); 3 The French Alternative Energies and Atomic Energy Commission (CEA), Biosciences and Biotechnologies Institute (BIAM), 30200 Bagnols-sur-Cèze, France; pisani.cedric@gmail.com (C.P.); odette.prat@cea.fr (O.P.); 4 NanoMedSyn, 15 Avenue Charles Flahault, 34090 Montpellier, France; morgane.daurat2@gmail.com (M.D.); afitz@hotmail.fr (A.D.S.); m.maynadier@nanomedsyn.com (M.M.); m.garcia@nanomedsyn.com (M.G.); 5 Laboratoire Innovations technologiques pour la Détection et le Diagnostic (Li2D), Service de Pharmacologie et Immunoanalyse (SPI), CEA, INRA, 30207 Bagnols-sur-Cèze, France; jean-charles.gaillard@cea.fr (J.-C.G.); jean.armengaud@cea.fr (J.A.); 6 Max Mousseron Biomolecule Institute of Montpellier (IBMM), 15 Avenue Charles Flahault, 34090 Montpellier, France; magali.gary-bobo@inserm.fr

**Keywords:** nanoparticles, safety, mesoporous silica, protein corona, internalization, adverse outcome pathways

## Abstract

Careful analysis of any new nanomedicine device or disposal should be undertaken to comprehensively characterize the new product before application, so that any unintended side effect is minimized. Because of the increasing number of nanotechnology-based drugs, we can anticipate that regulatory authorities might adapt the approval process for nanomedicine products due to safety concerns, e.g., request a more rigorous testing of the potential toxicity of nanoparticles (NPs). Currently, the use of mesoporous silica nanoparticles (MSN) as drug delivery systems is challenged by a lack of data on the toxicological profile of coated or non-coated MSN. In this context, we have carried out an extensive study documenting the influence of different functionalized MSN on the cellular internalization and in vivo behaviour. In this article, a synthesis of these works is reviewed and the perspectives are drawn. The use of magnetic MSN (Fe_3_O_4_@MSN) allows an efficient separation of coated NPs from cell cultures with a simple magnet, leading to results regarding corona formation without experimental bias. Our interest is focused on the mechanism of interaction with model membranes, the adsorption of proteins in biological fluids, the quantification of uptake, and the effect of such NPs on the transcriptomic profile of hepatic cells that are known to be readily concerned by NPs’ uptake in vivo, especially in the case of an intravenous injection.

## 1. Introduction

The growing interest of the scientific community for mesoporous silica nanoparticles (MSN) is particularly related to the degree of advanced sophistication that can be achieved in their design according to the objectives, in terms of properties or applications sought. Thanks to the silicon chemistry, these nanoparticles (NPs) have a promising potential to constitute a new generation of smart drug nanocontainers, due to their high stability, large surface area, tunable pore size, and abundant surface functionalization sites [1,2].

For these reasons, MSN are one of the most studied nanotechnologies for use as drug delivery systems. Because there are an increasing number of nanotechnology-based drugs, we can anticipate that regulatory authorities might adapt the approval process for nanomedicine products due to safety concerns, e.g., request a more rigorous testing of the potential toxicity of NPs. Understanding the interactions of NPs with biological systems is clearly multifactorial and complex. Nanoparticles display different shapes and sizes, and can be decorated with a variety of functionality. They have increased surface area-to-volume ratios that dramatically increase their reactivity. The miniaturization of materials to the nanoscale has seen emergent properties due to their ultralarge surface area. Their surface reactivity can, depending on the type of coating, cause different behavior and toxicological profiles. Thus, careful analysis of any new nanomedicine device or disposal should be undertaken to completely characterize the new products before application, so that we can help avoid any unintended side effects.

This perspective aims to present a series of biological assays performed to obtain an integrated overview of the safety of coated or non-coated magnetic MSN (Fe_3_O_4_@MSN). In vitro studies at the molecular and cellular level allow for rapid knowledge generation, and their results could be used as predictors before a validation phase, in terms of toxicological outcome in vivo. This two-stage approach could limit the extent, volume, and cost of animal testing. In this context, we propose to review the safety profile of MSN [3] and the panel of methodologies associated. Our interest focuses on MSN’s interaction with model membranes [4], the adsorption of proteins at their surface in biological fluids [5], the kinetics of internalization, and the effect of such NPs on the transcriptomic profile of hepatic cells [6] that are known to be readily concerned by NPs’ uptake in vivo, especially in the case of an intravenous injection.

## 2. Preparation of Magnetic Mesoporous Silica Nanoparticles

**Figure 1:**
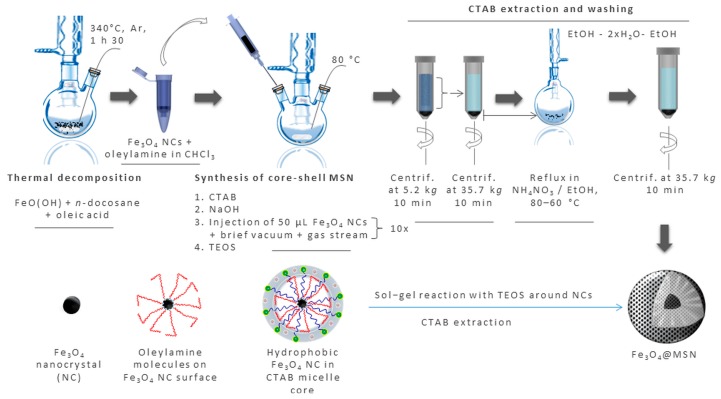
Schematic representation of the synthesis of bare magnetic mesoporous silica nanoparticles (Fe_3_O_4_@MSN). Firstly, Fe_3_O_4_ nanocrystals (NC) are obtained by thermal decomposition of FeO(OH). In another flask, cetyl trimethylammonium bromide (CTAB) micelles were obtained in alkaline water, at a temperature of 80 °C. Fe_3_O_4_ NC, after stabilization in oleylamine, were progressively added to the CTAB micelles, in 10 steps. After that, tetraethylorthosilicate (TEOS) has been added for sol–gel reaction and formation of Fe_3_O_4_MSN. Different washing steps were performed to extract CTAB surfactant from the pores.

Firstly, homogeneous NPs, reproducible synthesis, and extensive characterization are required to assess the toxicological profile of NPs. Before any biological assays, the NP synthesis has been designed to produce more potent NPs. Different aspects were even described as critical for NP safety, including biodegradability, surface properties (chemical composition, charge, hydrophilicity/hydrophobicity), and size. Mesoporous silica nanoparticles are biodegradable materials allowing drug release [7] while avoiding any accumulation and chronic toxicity, and which release silicic acid [8]. Dissolution of sol–gel-derived silica matrices occurs following two steps: an initial surface burst erosion followed by a slow bulk degradation [9]. The degradation rate and profile is dependent of the material composition [8], the production processes [10], the surface coating [11]**,** and the body fluids [12]. It has been shown that surfactant-extracted MSN are more quickly degraded than calcined ones or amorphous silica NPs [10] in simulated body fluids. Plus, calcination influences the surface properties and reactivity. This step allow the dehydration of the MSN surface, reducing the proportion of silanol groups, leading to siloxane groups [13]. The surface then becomes more hydrophobic, reducing the availability of the silanol groups to functionalization by covalent ligands or electrostatic coupling [14]. Mesoporous silica nanoparticles were synthesized with a magnetic core to follow them by magnetic resonance imaging (MRI) [15], to induce a heat-triggered drug release [16], and to separate them from complex media by magnetization. Synthesis of magnetic MSN was challenging to obtain a homogenous population of spherical Fe_3_O_4_@MSN, presenting a primary diameter of 100 nm, all containing a unique magnetic core, and without any step of calcination. The optimization of all these aspects was deeply described previously [3] (Figure 1).

## 3. Magnetic Separation for Corona Characterization

**Figure 2:**
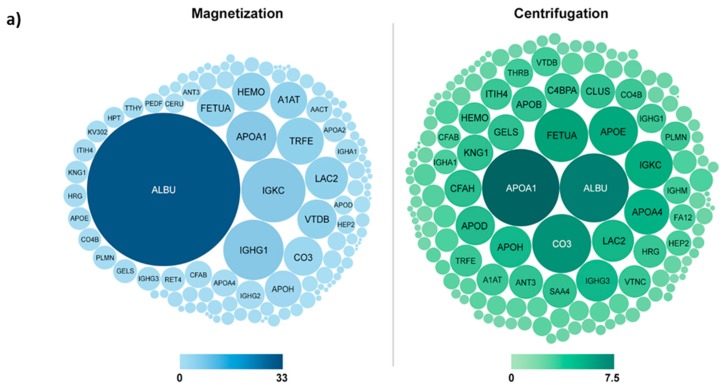

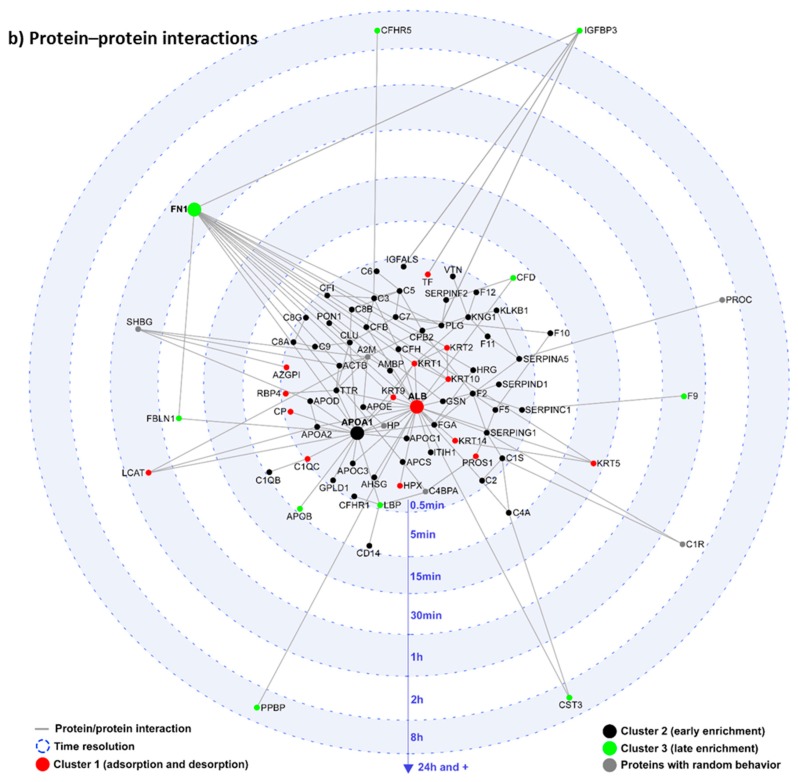
(**a**) Different proteomic profiles of the protein corona after Fe_3_O_4_@MSN separation by magnetization (blue) or centrifugation (green). Sizes and colors of human protein clues are proportional to their relative percentage within the corona. The 35 highest abundant proteins are labelled. The color scale unit is Normalized Spectral Abundance Factor (%). Reproduced from Pisani et al. 2017 [17] with permission from the Royal Society of Chemistry. (**b**) Protein–protein interactions are represented in a network developed using the NetworkAnalyst software [18,19], based on InnateDB [20]. The target represents the time scale (0.5 min to 7 days). Each protein (represented by its gene symbol) is placed according to its time of appearance within the corona. The colors indicate the cluster membership. The grey lines represent the protein–protein interactions. Proteins that have a lot of interactions with other proteins are represented by a larger visual cue. Reproduced from Pisani et al. 2017 [5] with permission from the Royal Society of Chemistry.

The use of magnetic nanoparticles allows for the efficient separation of the Fe_3_O_4_@MSN from biological media with a simple magnet [17]. Magnetic attraction of NPs is a useful method of separation to precisely characterize the protein corona, taking into account weak binding proteins, instead of very fast and drastic separation using centrifugation [17] (Figure 2a). This technique provides a true corona “interactome” of the MSN, with the characterization of the different protein–protein interactions around the NPs using next-generation shotgun proteomics [5] (Figure 2b). The timeline formation of the hard and the soft corona become accessible at low cost and relatively quickly, providing very interesting data for the development of future NPs.

## 4. Influence of Magnetic Mesoporous Silica Nanoparticles’ Coverage on Their Interaction with Proteins and Cell Membranes

**Figure 3:**
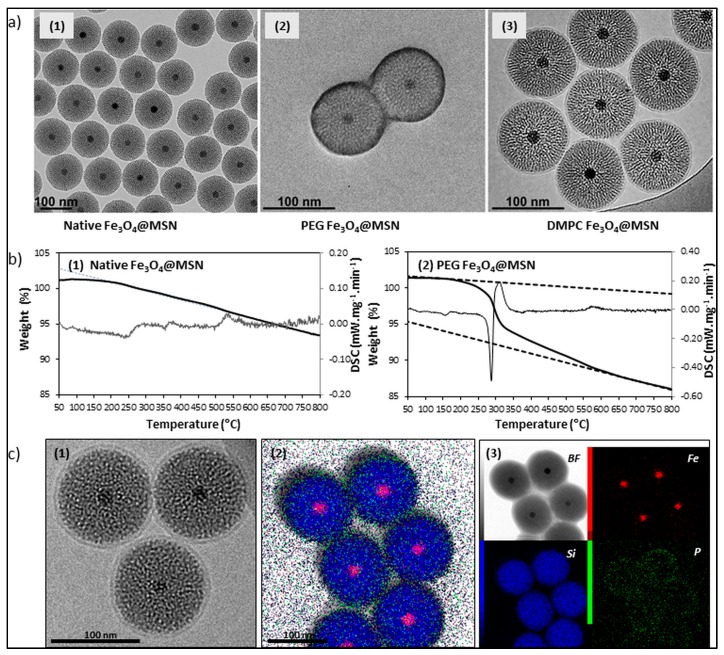
Characterization of native, polyethylene glycol (PEG)-grafted, and lipid-coated Fe_3_O_4_@MSN. (**a**) Transmission electron microscopy (TEM) imaging of (1) native Fe_3_O_4_@MSN, (2) PEG-grafted Fe_3_O_4_@MSN, and (3) lipid-coated Fe_3_O_4_@MSN with dimyristoyl phosphatidylcholine (DMPC) lipids, showing a primary diameter of 100 nm, with very homogeneous shape, porosity, and coverage. Reproduced from Pisani et al. 2017 [6], published under the Creative Commons Attribution (CC BY-NC-ND 4.0) license (https://creativecommons.org/licenses/by-nc-nd/4.0/). (**b**) Characterization of Fe_3_O_4_@MSN PEG-grafting, with thermogravimetric analysis (TGA)/dynamic scanning calorimetry (DSC) spectra of (1) pristine Fe_3_O_4_@MSN and (2) PEG–Fe_3_O_4_@MSN. (**c**) Imaging of magnetic Fe_3_O_4_@MSN core–shell particles after incubation with DMPC small unilamellar vesicles (SUVs) (1). All the MSN are covered with a complete lipid bilayer, having a thickness of 5 nm. Three lipid-coated MSN are zoomed in on for a better observation of the lipid bilayer. (2) Scanning transmission electron microscopy (STEM) images of DMPC Fe_3_O_4_@MSN: DMPC Fe_3_O_4_@MSN overlay of TEM black field (BF), iron (Fe), silica (Si), and phosphorus (P) element cartography. (3) Each element is separately presented. The iron core localizes at the center of the silica nanoparticles and phosphorus is localized around the silica shell of the Fe_3_O_4_@MSN particles. (**b**) and (**c**) are reproduced and adapted from Nyalosaso et al. 2016 [3] with permission from the Royal Society of Chemistry.

To reduce the formation of the protein corona at the NPs’ surfaces, NPs are generally covered by different layers. Numerous reported studies demonstrate that a polyethylene glycol (PEG) coating presents several advantages, like colloidal stability, inertia in biological media, and higher circulation time of NPs [11,21]. Another strategy consists of the deposition of a phospholipid bilayer on the inorganic NPs’ surface, in order to create a biomimetic surface [22,23]. Fusion of liposomes to a spherical, high surface area, nanoporous silica core improves capacity, selectivity, and stability of NPs, and enables their targeted delivery and controlled release within the targeted cells [22,24,25]. Moreover, these two types of coverage can be easily applied to inorganic NPs [3,26,27] (Figure 3). Different strategies were employed for the functionalization of the NPs [28]. Concerning the lipid coating of MSN, this can be achieved by spontaneous adsorption of small unilamellar vesicles on the silica surface of NPs in suspension [27], or by thin-layer lipid rehydration in presence of the NPs [28,29]. Various parameters were investigated for their influence on effective NP coverage, such as buffer composition (pH, ionic strength) [30], lipid/NP ratio [31], temperature [32], or NP size [26]. Characterization of the lipid coating of the NPs is often done by cryogenic transmission electron microscopy (TEM), dynamic light scattering (DLS), zeta potential (ZP), or dynamic scanning calorimetry (DSC). These methods allow qualitative characterization of the NPs’ coating, while quantitative methods can also be used, such as inorganic phosphorus dosage [30] or elemental analysis [29]. Preparation of PEG-grafted MSN is generally performed by direct addition of PEG-silane at the end of the NPs synthesis [33]. The silane groups are then able to condense with the silica surface of the NPs [34]. In this case, PEG is covalently bound to the NPs’ surface [35]. Characterization is also frequently done by TEM imaging, DLS, or ZP [36]. Fourier-transformed infrared (FTIR) spectroscopy [35], DSC, or thermogravimetric analysis (TGA) allow deeper characterization [34]. It should be noted that combinations of surface decoration strategies are now described, leading to highly sophisticated nanocarriers that combines stealth properties, targeting, and controlled or triggered release [37]. For example, PEG–lipid assemblies are used for the functionalization of MSN [38].

**Figure 4:**
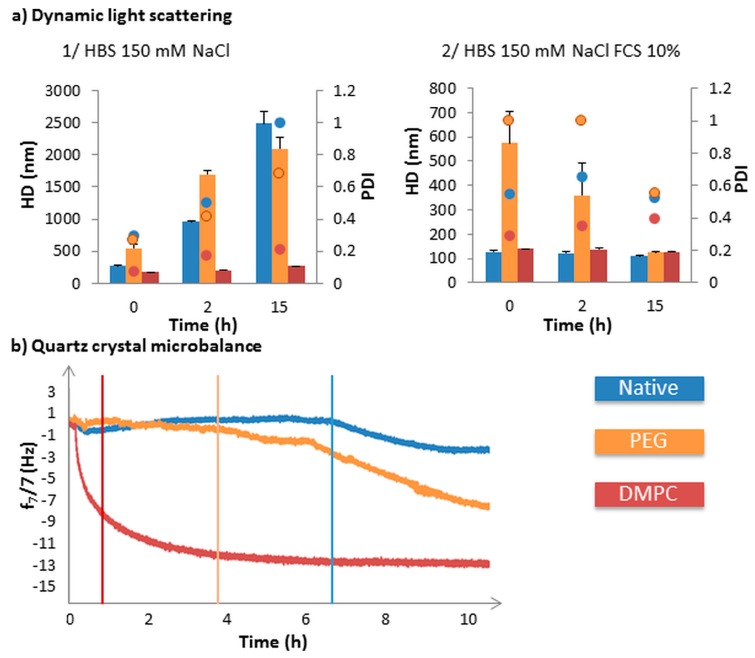
Characterization of native and coated Fe_3_O_4_@MSN behavior in suspension in complex media, with or without proteins. (**a**) (1) and (2): Hydrodynamic diameter (HD) and polydispersity index (PDI), represented respectively by bars and dots, for native (blue), polyethylene glycol (PEG) (orange) and dimyristoyl phosphatidylcholine (DMPC) (red) Fe_3_O_4_@MSN in (x) HEPES buffered saline (HBS) 150 mM NaCl (pH 7.4) and (y) HBS 150 mM NaCl (pH 7.4) containing 10% fetal calf serum (FCS). (**b**) Quartz crystal microbalance with dissipation (QCM-D) frequency sensorgram following the interaction between nanoparticles and the egg phosphatidyl choline (EPC)-supported lipid bilayer (SLB). Native (blue), PEG (orange), and DMPC (red) Fe_3_O_4_@MSN were flowed into HBS 150 mM NaCl 10% SCF medium on the top of EPC SLB, at a concentration of 0.25 mg mL^−1^ of nanoparticles. After adding Fe_3_O_4_@MSN into the medium on the top of the EPC SLB for 15 min, the flow was stopped for 10 h. The results on the variations of frequency are presented after the offset of the lipid bilayer formation. Reproduced and adapted from Rascol et al. 2017 [4], published under the Creative Commons Attribution (CC BY) license (http://creativecommons.org/licenses/by/4.0/).

The coating of Fe_3_O_4_@MSN by polymers or lipid bilayers was shown to influence the colloidal stability and interaction with proteins, model membranes, and cells in vitro [3]. Lipid bilayers allowed colloidal stability of MSN in a high ionic strength medium, in comparison to native or PEG-coated MSN (Figure 4a1). Native NPs were stabilized (meaning colloidal stability) in the presence of proteins by the formation of the corona, while PEG-coated MSN were very slowly stabilized by the presence of the proteins, due to the MSN’s low adsorption of those proteins (Figure 4a2). The formation of a protein corona appeared to reduce the interaction between MSN and model membranes composed of an egg phosphatidylcholine (EPC)-supported lipid bilayer (SLB) as was observed using quartz crystal microbalance with dissipation (QCM-D) [4] (Figure 4b). Lipid-coated MSN were rapidly deposited on the top of the lipid bilayer, while PEG-coated MSN deposited slowly, and the native ones remained suspended in the medium.

## 5. Magnetic Mesoporous Silica Nanoparticles’ In Vivo Toxicity and In Vitro Mechanisms

**Figure 5:**
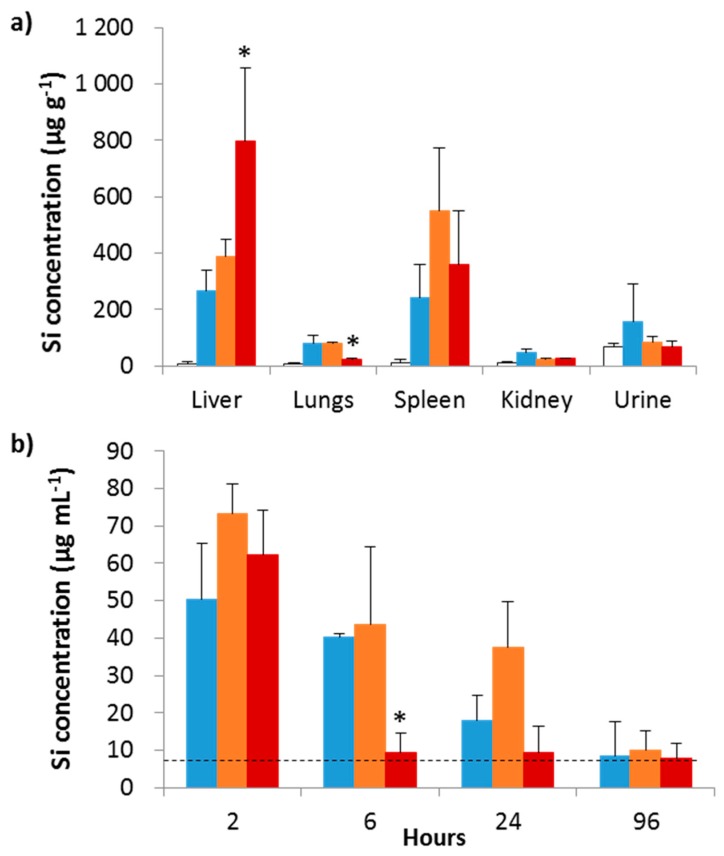
Biodistribution of Fe_3_O_4_@MSN in mice. (**a**) Quantification of silicon in different organs four days after injection. Inductively coupled plasma–mass spectrometry (ICP–MS) was used after acid digestion to quantify the silicon in the liver, lungs, spleen, kidneys, and urine four days after intravenous injection of native (blue), polyethylene glycol (PEG) (orange) and dimyristoyl phosphatidylcholine (DMPC) (red) Fe_3_O_4_@MSN at a concentration of 40 mg kg^−1^ in comparison to control mice (white). (**b**) Nanoparticle level in blood. The silicon levels in blood were measured 2, 6, 24 h, and 4 days after intravenous injection of native (blue), PEG (orange), and DMPC (red) Fe_3_O_4_@MSN at a concentration of 40 mg kg^−1^. The dashed line indicates the silicon level found in blood of control mice. For this experiment, 20 mice were divided into four groups of five animals. The values of the histograms represent the mean ± standard deviation (SD) of values of each animal of a group. * *p* < 0.05 indicates that a group is statistically different from all other groups treated with nanoparticles. Reproduced and adapted from Rascol et al. 2017 [4], published under the Creative Commons Attribution (CC BY) license (http://creativecommons.org/licenses/by/4.0/).

Functionalized Fe_3_O_4_@MSN with lipid bilayers or PEG polymers were administered to mice by intravenous injections, at a dose of 40 mg kg^−1^, in order to compare their distribution and toxicity to bare Fe_3_O_4_@MSN [4]. Magnetic mesoporous silica nanoparticles were quantified by inductively coupled plasma–mass spectrometry (ICP–MS) analysis in the different organs obtained from sacrificed mice four days after injection (Figure 5a). All of the Fe_3_O_4_@MSN, functionalized or not, accumulated in the liver and spleen. However, lipid bilayer-coated Fe_3_O_4_@MSN accumulation was largely higher in the liver; these nanoparticles were found in a lower concentration in the lungs, and were cleared more quickly from the blood than the bare and PEG-grafted Fe_3_O_4_@MSN. PEG-grafted Fe_3_O_4_@MSN were always found in the blood 24 h after injection (Figure 5b). However, it was demonstrated that none of the Fe_3_O_4_@MSN caused toxicity to liver, kidney, and spleen tissues at the administered doses [4]. Moreover, no immunotoxic effect was observed at the animal level.

**Figure 6:**
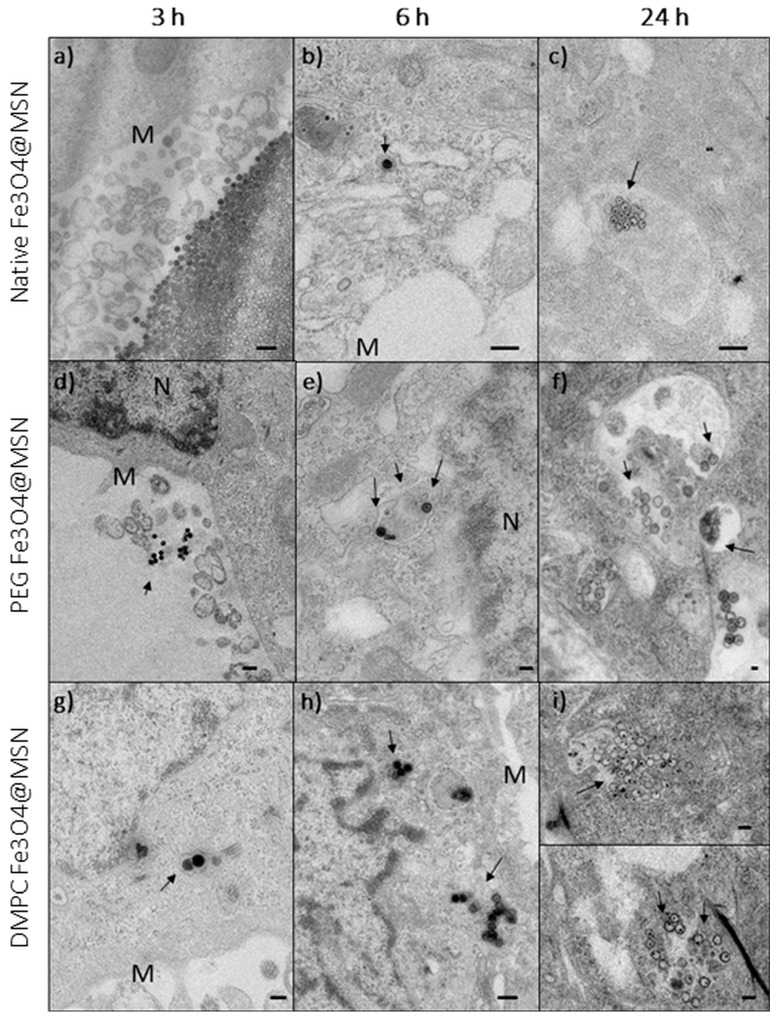
Transmission electron microscopy imaging of HepG2 cells exposed for 3, 6, and 24 h at 50 μg mL^−1^ for (**a**–**c**) native, (**d**–**f**) polyethylene glycol (PEG)-coated, or (**g**–**i**) dimyristoyl phosphatidylcholine (DMPC)-coated Fe_3_O_4_@MSN. The nanoparticles are indicated by arrows, near the cell membrane (M) or the nucleus (N). Reproduced from Rascol et al. 2017 [4], published under the Creative Commons Attribution (CC BY) license (http://creativecommons.org/licenses/by/4.0/).

**Figure 7:**
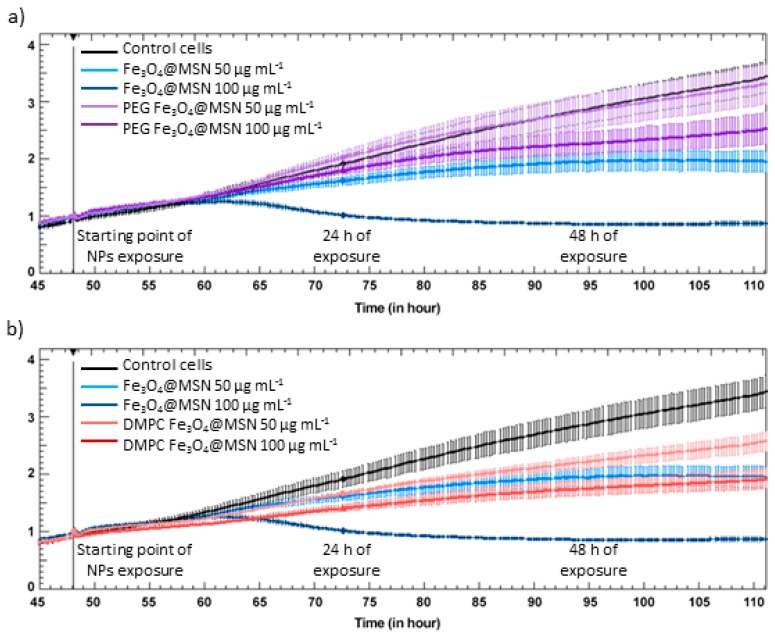
xCELLigence experiment. Real-time cell index (CI) monitoring of HepG2 cells (*n* = 3) exposed to 50 and 100 mg mL^-1^ of pristine, polyethylene glycol (PEG)-coated, and dimyristoyl phosphatidylcholine (DMPC)-coated Fe_3_O_4_@MSN. (**a**) Pristine Fe_3_O_4_@MSN versus PEG-coated Fe_3_O_4_@MSN. (**b**) Pristine Fe_3_O_4_@MSN versus DMPC-coated Fe_3_O_4_@MSN. Reproduced and adapted from Nyalosaso et al. 2016 [3] with permission from the Royal Society of Chemistry.

Different techniques were combined to investigate the potential toxic effects of these Fe_3_O_4_@MSN, covered or not covered by PEG or lipid bilayers at the cellular level. First, in vitro analyses were performed by exposure of two liver cell lines to the different particles, the rational for choosing the cell lines being dictated by the preferential liver uptake of MSN. On one hand, HepG2 is a very frequently human hepatocarcinoma cell line studied for in vitro evaluation of anticancer therapy. On the other hand, HepaRG is a human hepatocarcinoma cell line which can be differentiated in vitro in hepatocyte-like colonies surrounded by clear primitive biliary cells [39,40]. The cell uptake of the Fe_3_O_4_@MSN was faster for those that were coated with DMPC than for the native ones, and slower for the PEG-grafted Fe_3_O_4_@MSN. This was observed by TEM cell imaging after exposure of HepG2 (Figure 6) and HepaRG cell lines to Fe_3_O_4_@MSN [6]. This observation was in accordance with the rapid deposition of lipid-coated MSN on membrane models [4]. Moreover, the effect of Fe_3_O_4_@MSN on HepG2 and HepaRG cell lines was also investigated by cell impedance [3,6]. Impedance measurement of HepG2 cells showed a greater decrease in cell impedance by exposure to native Fe_3_O_4_@MSN than by exposure to the lipid-coated ones, and lastly, by exposure to the PEG-grafted Fe_3_O_4_@MSN [3,6] (Figure 7). Thus, impedance measurement represents an important metric to document.

**Figure 8:**
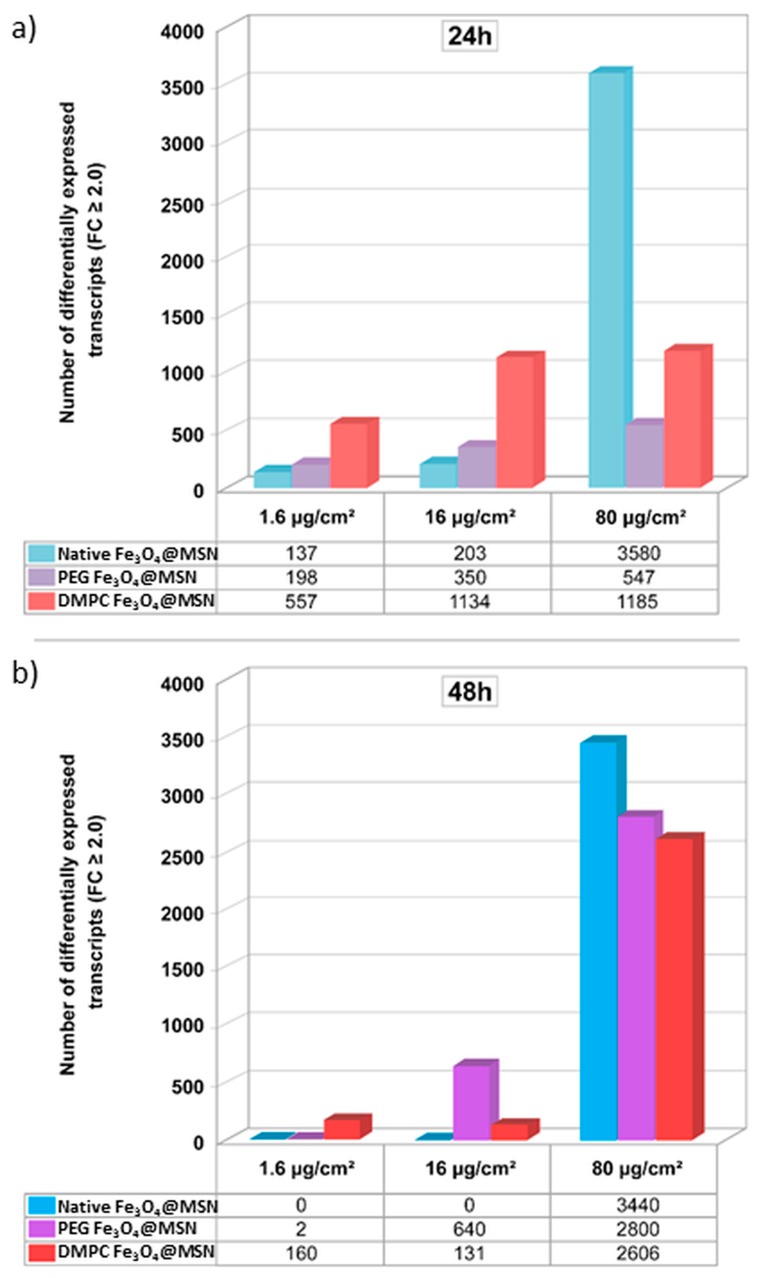
Time- and dose-dependent effects of exposure to Fe_3_O_4_@MSN on the number of significantly differentially expressed genes. HepaRG cells were exposed to 1.6, 16, and 80 µg cm^−2^ pristine, polyethylene glycol (PEG)-, and dimyristoyl phosphatidylcholine (DMPC)-coated Fe_3_O_4_@MSN for 24 (**a**) or 48 (**b**) h. After extraction and labeling, RNA was hybridized to a human oligonucleotide microarray (6 × 60 k Agilent V3 SurePrint). Bars represent the number of differentially expressed transcripts after statistical analysis, using Genespring GX13 software (Agilent), and with a *p*-value < 0.05 and a fold-change (FC) ≥ 2. Reproduced from Pisani et al. 2017 [6], published under the Creative Commons Attribution (CC BY-NC-ND 4.0) license (https://creativecommons.org/licenses/by-nc-nd/4.0/).

**Figure 9:**
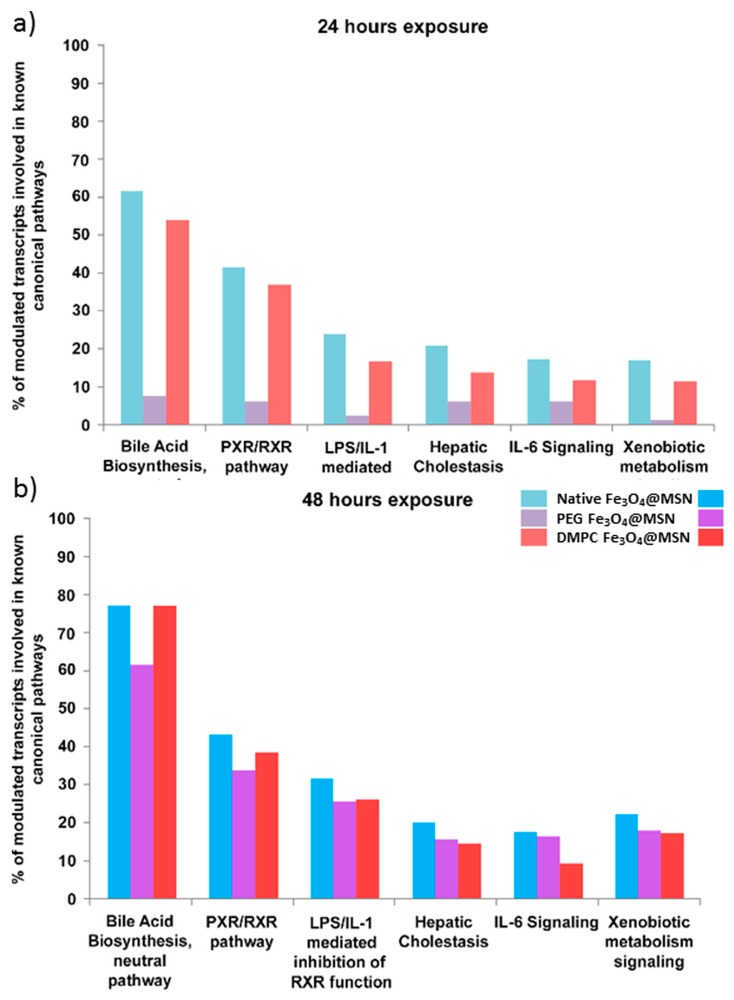
Canonical pathways elicited by each Fe_3_O_4_@MSN (80 mg cm^−2^). The percentage of modulated transcripts of our datasets belonging to six major altered canonical pathways after (**a**) 24 and (**b**) 48 h exposure to Fe_3_O_4_@MSN. These pathways were all significant according to a Fisher’s statistical test (*p*-value < 0.05), revealed with Ingenuity^®^ Pathway Analysis (IPA^®^, QIAGEN). Reproduced from Pisani et al. 2017 [6], published under the Creative Commons Attribution (CC BY-NC-ND 4.0) license (https://creativecommons.org/licenses/by-nc-nd/4.0/).

To get more insights into the molecular mechanisms explaining the biological effects of these Fe_3_O_4_@MSN in vitro, high-throughput transcriptomic assays were carried out [6]. Changes in the expression of thousands of genes were monitored for the HepaRG cell line after an exposure to different doses of the native, PEG-grafted, and lipid-coated Fe_3_O_4_@MSN. A transient change in the expressed gene profile has been observed for the lower doses between 24 and 48 h (Figure 8a,b, respectively), indicating that 16 µg cm^−^² (or 60 μg mL^−1^) could be the limit of biocompatibility for all of these Fe_3_O_4_@MSN. Slight differences could be observed depending on Fe_3_O_4_@MSN surface modifications. However, whatever the type of Fe_3_O_4_@MSN, 80 µg cm^−^² (or 300 μg mL^−1^) represents a concentration with strong and lasting adverse effects. At this concentration, initial molecular events and major pathways of toxicity elicited by these Fe_3_O_4_@MSN were easily identified by the methodology used. The hepatic cholestasis adverse pathway was triggered by a strong inhibition of the bile salt export pump transporter protein BSEP (gene ABCB11), responsible for intrahepatic accumulation of cytotoxic bile acids [6] (Figure 9).

## 6. Conclusions

This review highlighted the results of biological evaluation obtained on bare, PEG-grafted, and lipid-coated Fe_3_O_4_@MSN, presenting the same physical and chemical characterizations for all safety investigation performed. Some results presented here were previously discussed in other studies, such as NP aggregation at high ionic force, adsorption of proteins to the surface, or NP accumulation in the liver and spleen. However, this is the first time that all these aspects have been investigated, using different techniques to compare NPs with different surface properties, and the results have been reported as a whole here. This pioneering work allows for linking the different reported data and methodologies to a more in-depth analysis of the biological effects of Fe_3_O_4_@MSN at the animal, cellular, and molecular levels. This compendium is an example of integrated investigation of the safety of innovative technologies for biomedical applications. This multi-technology approach paves the way for the future trend of safety recommendations, including methodologies that are at the forefront of their disciplines while being rather cost- and time-effective.
